# Analysis of heterogeneous genomic samples using image normalization and machine learning

**DOI:** 10.1186/s12864-020-6661-6

**Published:** 2020-12-21

**Authors:** Sunitha Basodi, Pelin Icer Baykal, Alex Zelikovsky, Pavel Skums, Yi Pan

**Affiliations:** 1 Department of Computer Science, Georgia State University, 25 Park Place NE, Atlanta, GA 30303 USA; 2 The Laboratory of Bioinformatics, I.M. Sechenov First Moscow State Medical University, Moscow, 11991 Russia

**Keywords:** Next-generation sequencing data, Image normalization, Staging HCV infections, Outbreaks investigations, Clustering

## Abstract

**Background:**

Analysis of heterogeneous populations such as viral quasispecies is one of the most challenging bioinformatics problems. Although machine learning models are becoming to be widely employed for analysis of sequence data from such populations, their straightforward application is impeded by multiple challenges associated with technological limitations and biases, difficulty of selection of relevant features and need to compare genomic datasets of different sizes and structures.

**Results:**

We propose a novel preprocessing approach to transform irregular genomic data into normalized image data. Such representation allows to restate the problems of classification and comparison of heterogeneous populations as image classification problems which can be solved using variety of available machine learning tools. We then apply the proposed approach to two important problems in molecular epidemiology: inference of viral infection stage and detection of viral transmission clusters using next-generation sequencing data. The infection staging method has been applied to HCV HVR1 samples collected from 108 recently and 257 chronically infected individuals. The SVM-based image classification approach achieved more than 95% accuracy for both recently and chronically HCV-infected individuals. Clustering has been performed on the data collected from 33 epidemiologically curated outbreaks, yielding more than 97% accuracy.

**Conclusions:**

Sequence image normalization method allows for a robust conversion of genomic data into numerical data and overcomes several issues associated with employing machine learning methods to viral populations. Image data also help in the visualization of genomic data. Experimental results demonstrate that the proposed method can be successfully applied to different problems in molecular epidemiology and surveillance of viral diseases. Simple binary classifiers and clustering techniques applied to the image data are equally or more accurate than other models.

## Background

Currently, viral epidemics continue to be critical public health issues. Many emerging and long-standing epidemics are associated with small (~ 10 kilobases long) positive-sense single stranded RNA virus, such Human Immunodeficiency Virus (HIV), Hepatitis C virus (HCV), Zika virus (ZIKV) and dengue virus (DENV). The paramount feature of these viruses is their extremely high mutation rate caused by error-prone replication, which can be as high as 10^-4^ mutations per site per replication cycle [1], thus resulting in generation of all possible single point mutations in each infected individual every day. As a result, RNA viruses exist in infected hosts as highly heterogeneous populations of genomic variants usually referred to as *viral quasispecies*. Intra-host and inter-host evolution of viral quasispecies is a complex phenomenon de defined by numerous factors such as virulence, infectivity, drug resistance, immune escape, transmission rates, behavorial patterns and otherphenotypic and epidemiological numerous factors such as virulence, infectivity, drug resistance, immune escape, transmission rates, behavorial patterns and other phenotypic and epidemiological features, which plays crucial role in disease progression and outcome of infection [2–6]. Challenges associated with understanding complex quasispecies evolution attracted many researchers in different domains, including virology, epidemiology, population genetics and systems biology.

Analysis of heterogeneous viral populations is one of the most challenging bioinformatics tasks owing both to the complexity of the underlying algorithmic problems and features and sheer amount of data [7, 8]. These challenges became especially complicated in the recent decade with the advent of high-throughput sequencing (HTS), which has now become a major tool for viral research, allowing to sample viral populations at unprecedented depth [9–15]. Modern computational virology continues mostly to rely on classical approaches, which include sequence analysis, phylogenetics/phylodynamics and structural bioinformatics [8, 16]. In the recent years, these approaches started to be complemented with the network analysis [17–19].

Significant number of computational molecular epidemiology problems could be defined using phylogenetics or clustering-based objective. These problems include inference of transmission clusters, detection of co-infections, therapy outcome prediction, infection staging and other research and medical questions. Such problems could be tackled by powerful methods of machine learning and deep learning. It should be expected that in the near future, in accordance with the general trend in AI and Computer Science research, machine learning and deep learning techniques will be utilized in viral research on a much wider scale.

In order to employ machine learning for viral studies, quasispecies populations should be transformed into feature vectors from a multidimensional euclidean space. Several encoding schemes have been used in the literature for transforming biomedical data into numerical data for machine learning [20]. However, the existing methods face significant challenges when applied to viral genomic data. These challenges are associated with extremely high heterogeneity of intra-host viral populations, sequencing errors and sampling biases, robustness to noise and difficulty of selection of relevant sets of features.

### Contribution

In this work, we propose a novel method converting genomic data into images, which are then used for classification and clustering. The new approach allows to utilize a well-developed machine learning methodology from the domain of image processing in genomic analysis. The proposed scheme provides the data structure for the representation of intra-host population structure which is compact, easily adjustable, robust to technological noise and sampling bias, preserve structural properties of populations and can be used for a variety of classification problems, where machine learning is applicable.

We validated our approach by applying image processing techniques to two important molecular epidemiology problems. The first problem is the HCV infection staging, i.e. distinguishing between recent and chronic infections using viral sequences sampled by next-generation sequencing (NGS). It is known that in 80% of untreated cases HCV infection turns into a chronic infection leading to severe health problems such as liver cirrhosis and hepatocellular carcinoma (a form of liver cancer). HCV infection often does not manifest any clinical symptoms in its early stages, which impedes the timely diagnosis of disease. Furthermore, currently there are no diagnostic assays to determine the stage of HCV infection. Therefore, distinguishing recently infected patients from chronically infected patients using non-invasive methods such as analysis of genomic data would be highly important both for personalized therapeutic purposes and for epidemiological surveillance; e.g., for detection of incident HCV cases.

The second problem is the detection of outbreaks using NGS data. In molecular epidemiology, it is common to utilize the observation that viral populations from the same outbreak are genetically related. Thus, measures of genetic relatedness could be used as a predictor for epidemiological relatedness [21–23]. In other words, this problem could be considered as the problem of clustering of intra-host viral populations. Until recently, most available tools for outbreak investigations analyzed only a single representative sequence per population (usually consensus sequence) [21, 23]. Although several recently published tools allow to take into account entire intra-host populations [18, 19, 22, 24], the problem of comparison and clustering of viral populations still remains challenging [25].

We demonstrate that classification and clustering techniques based on normalized image representations of intra-host viral populations allow to solve these two problems with high accuracy.

## Methods

### Data collection

Intra-host HCV populations sampled by sequencing of a highly heterogeneous genomic region (HVR1) are analyzed. The analyzed region of length 264 bp, which in-cludes HVR1, has been sequenced using the GS FLX System and the GS FLX Titanium Sequencing Kit (454 Life Sciences, Roche, Branford, CT). Obtained sequences were processed using the error correction and haplotyping algorithm KEC [26], and the obtained haplotypes were aligned using Muscle [27]. The data [16, 28] used for classification of intra-host HCV populations as recent and chronic consists of 365 NGS samples, including 108 datasets corresponding to recently infected hosts and 257 datasets belonging to chronically infected hosts. Recent samples either belong to patients with the known times since seroconversion, or to the collection of HCV outbreaks, where epidemiological investigations revealed that secondary cases were infected within few months from the dates of sample collection, thus allowing to classify them as recently infected. Chronic samples are obtained from several molecular surveillance studies. For clustering and identication of outbreaks, we use the benchmark dataset [18, 19, 22] that consists of HCV intra-host populations collected from 335 infected individuals in 2008-2013. Of these, 142 HCV samples belong to epidemiologically curated outbreaks involving from 2 to 19 patients, while the remaining datasets are epidemiologically isolated cases.

### Sequence image normalization

**Fig. 1:**
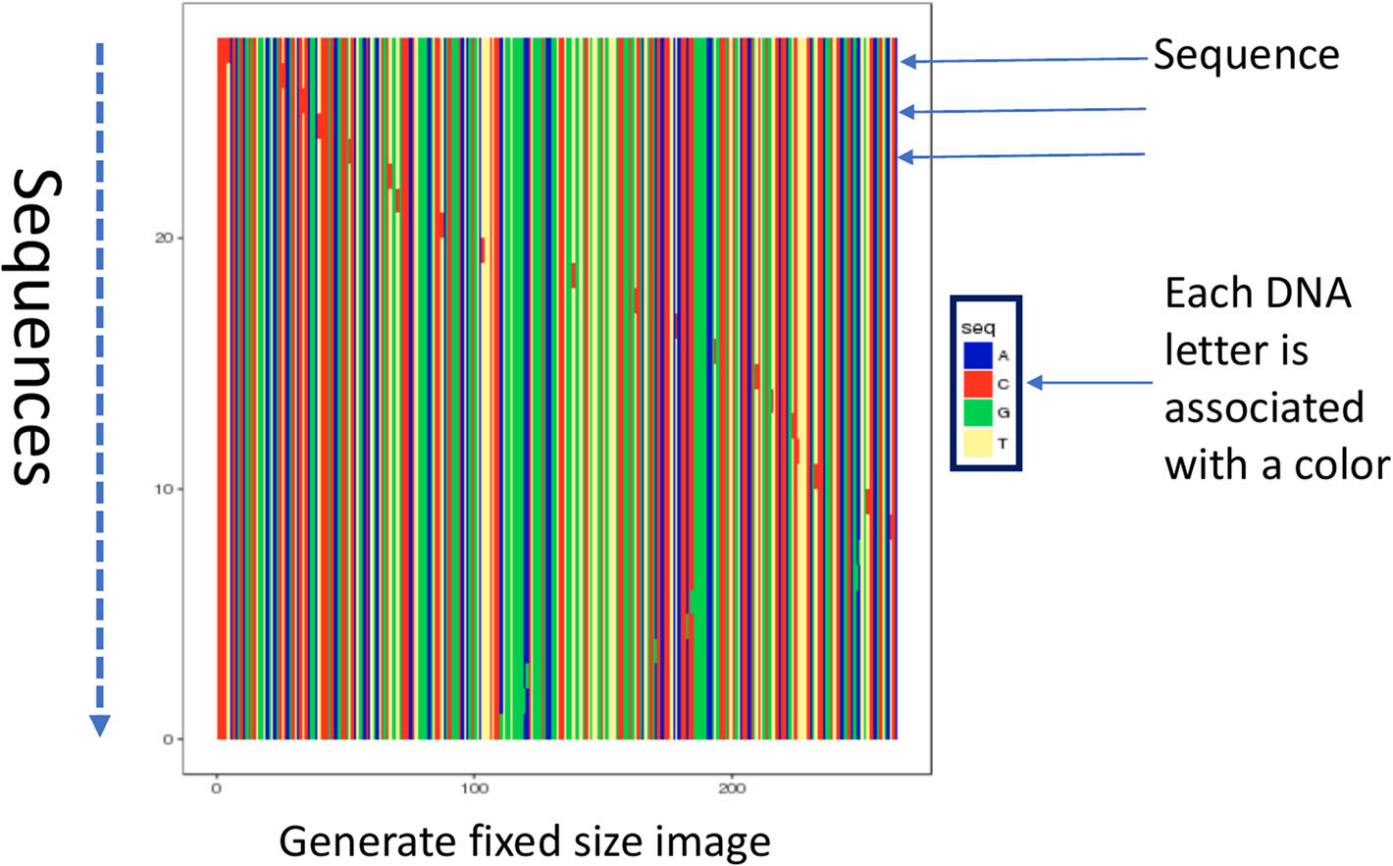
Generation of fixed size image

We transform sequence data into an image by the preprocessing method further referred to as Sequence Image Normalization. We assume that sequences are aligned and ordered by their counts, with sequences of the same counts being sorted lexicographically. Next, each symbol l 2 f^0^A^0^;^0^ C^0^;^0^ T^0^;^0^ G^0^;^0^ ^0^g is associated with a particular color thus transforming the sequence alignment into an image. Finally, the images corresponding to different infected hosts are normalized by transforming them into fixed size images. The colors to represent nucleotides are selected from the set of colors of higher variation in order to simplify identification of discriminative size images. The colors to represent nucleotides are selected from the set of colors of higher variation in order to simplify identification of discriminative features characterizing particular intra-host populations. Fig. 1 demonstrates an example of sequence image normalization output. Normalized images thus allow to captures entire viral population structure using a single data representation independent of the number of sequences and with minimum loss of existing data or introduction of artificial data.

**Fig. 2:**
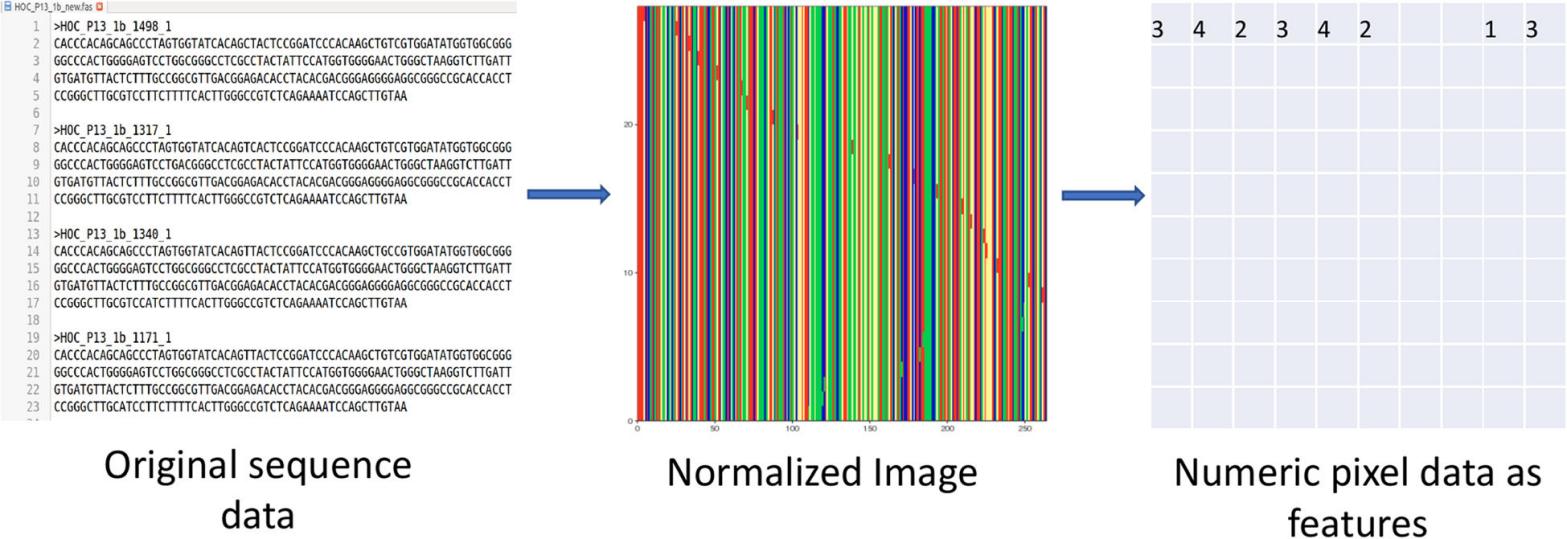
Pipeline of sequence image normalization of a fasta file

Raw pixel data of generated images are used as features to train machine learning models for the consecutive analysis, as demonstrated in Fig. 2. The number of features depends on the image resolution: each image of the resolution x× y corresponds to x× y × 3 feature vector, with each pixel having 3 RGB components. In our experiments, sequence datasets have been analyzed for different resolutions ranging from 50 × 50 to 550 × 550 with the step size of 50 in each dimension. Results were generated using resolution 480 × 480 at which both models performed most accurately.

### Classification of recent and chronic HCV infections

**Fig. 3:**
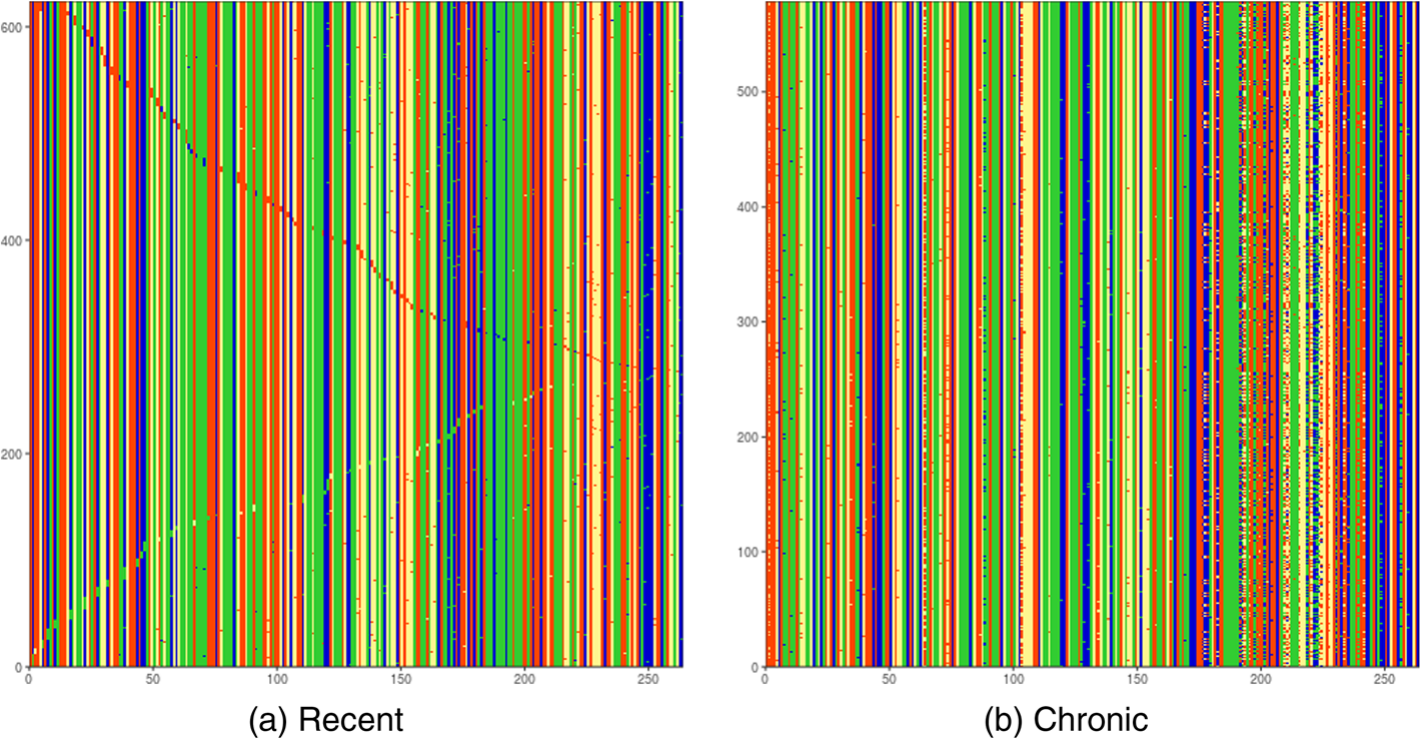
Examples of normalized images of intra-host populations from (a) recent HCV infection and (b) chronic HCV infection

Identification of HCV infection stages is considered as a binary classification problem. Fig. 3 shows typical normalized images of HCV populations from recent and chronic infections. Visual inspection of images allows for identification of typical patterns associated with both classes - images of recent infection have pronounced diagonal lines while chronic images are choppy.

Images corresponding to intra-host viral populations have been labeled based on the stage of infection as recent or chronic and used to train the following machine learning classification models: Stochastic Gradient Descent (SGD), Decision Tree, Gaussian Naive Bayes (Gaussian NB), Linear Support Vector Machine (Linear SVM), Random Forest and *k*-Nearest Neighbours (*k*NN). We used models’ implementations from python *scikit-learn* library [29]. Different SVM kernels have been explored of which SVM with linear kernel produced the best results. In linear SVM model, there is a regularization parameter* c* which helps in generalizing the model by controlling testing and training errors. In this model, grid search is performed on *c* values in the range [-2, 20]. For kNN models, we selected the best model among the models with euclidean and manhattan metrics and with *k* from the range [3, 20]. For random forest, the best model has been chosen by performing grid search on the number of trees in the range [10, 100].

Trained classifiers have been validated based on their accuracy, area under the curve (AUC), precision, and recall. Accuracy (Acc) is defined as the proportion of test cases correctly classified, as either recent or chronic. Precision (Prec) measures the fraction of the correctly classified populations within each predicted infection class, while recall (Rec) measures the fraction of the true recent or chronic populations that are correctly predicted. Validation has been performed via stratified 10-fold cross-validation. Specifically, in addition to the standard 10-fold cross-validation, we employ "leave-one-outbreak-out" cross-validation and random undersampling methods to balance the datasets. In our current data, some of the samples come from the same HCV outbreak. Such samples are close to each other by their nucleotide composition, thus their presence may lead to over-fitting of any particular method. In "leave-one-outbreak-out" cross-validation, data from each of these outbreaks was used in the validation set, while other samples are used in the training sets. Random undersampling has been performed to balance the difference in sizes of datasets of recent and chronic hosts. In this method, chronic dataset size is reduced by random subsampling to match the recent dataset size.

### Clustering of intra-host viral populations from outbreaks

We cluster images representing intra-host viral populations into transmission clusters using standard clustering algorithms {agglomerative hierarchical clustering, *k*-means clustering and mini-batch *k*-means clustering. As before, we used models’ implementations from python *scikit-learn* library [29]. Several distance measures have been employed, including euclidean, manhattan and cosine metrics. Hierarchical clustering has been executed using complete, average and ward linkage approaches.

Normalized Mutual Information (NMI) [30], homogeneity [31] and completeness [31] scores as used as metrics to analyze the clustering performance. These measures evaluate the assigned cluster labels after clustering compared to the actual cluster class label of each intra-host viral population. Homogeneity score measures if the all members of a cluster actually belong to one cluster class label, while the completeness scores measure if all the members of an actual cluster class label are grouped into the same cluster. NMI measures the mutual information shared between the individuals in the clusters. All these measures range from 0 to 1 and the values closer to 1 refer to better clustering efficiency. To evaluate the effectiveness of the normalization method in detecting relatedness between any pair of samples, we compute AUROC (Area under ROC curve) is computed (as done in [18]). Viral populations taken from the same outbreak are considered as genetically related, otherwise as unrelated. There are 55,945 pairs of samples, and 479 of them are related. After computing the distances between each pair of samples, all the pairs crossing a threshold value are considered as related. To compute AUROC curve, false-postive rate (FPR) and true-postive rate (TPR) are measured by modifying the threshold starting from the best threshold value where there are no false positives.

## Results

### Classification of infection stages

**Fig. 4:**
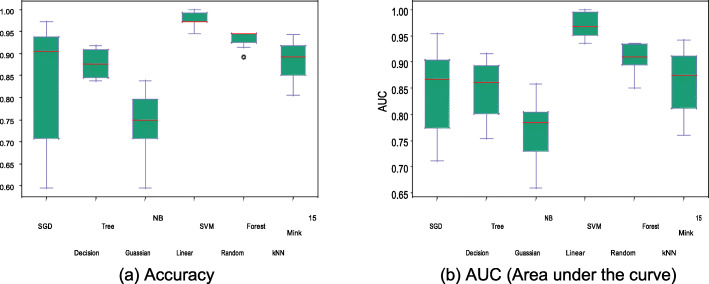
Accuracy and AUC (Area Under the Curve) for several simple classification methods after training based on the normalized image data

**Fig. 5:**
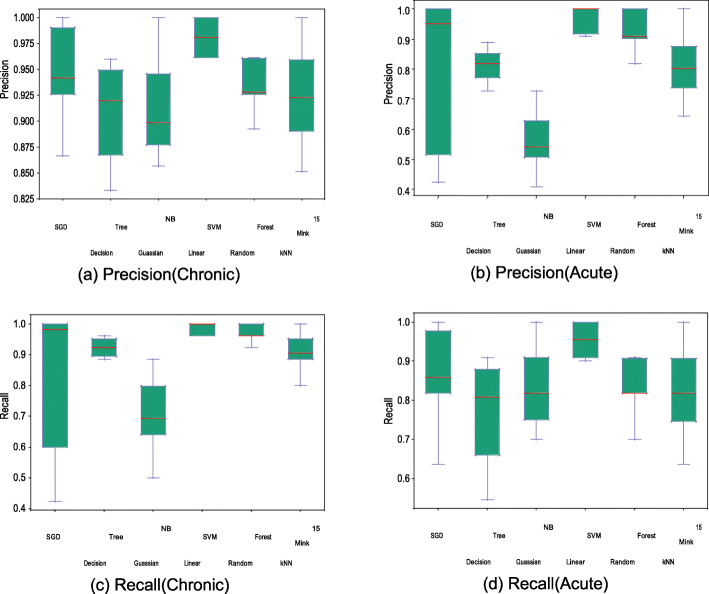
Precision and Recall comparisons of several simple classification methods after training based on the image data generated using sequence image preprocessing method

Stratified 10-fold cross-validation has been initially performed to analyze the performance of several classification methods trained using the normalized image data. Fig. 4 shows accuracy and AUC of the best models for each of the methods using box plots, with the average metrics being indicated by red line. Linear SVM demonstrated superior performance compared to all other models, with an average accuracy of 97.545% and low accuracy variance. Other models with the exception of Gaussian NB have accuracy greater than 85%, thus exceeding accuracy of existing methods, which are primarily based on feature extraction methods (see Comparison with previous methods subsection). Accuracy metric alone cannot define performance of the model as it needs to achieve higher precision and recall metrics for each infection type as well. Fig. 5a-d demonstrate the precision and recall metrics for chronic and recent samples separately. As before, linear SVM achieves the best performance over all other models with an average precision and recall values of 98.11 and 98.45% for chronic populations and 96.52 and 95.36% for recent populations, respectively. This model also has low variance across the values obtained from all the folds. Noticeably, other models with the exception of Gaussian NB also achieve more than 80% values for these metrics.

Linear SVM model has been analyzed further with leave-one-outbreak-out and random undersampling validation combined with 10-fold cross-validation. Table 1 shows the results of these methods compared to the standard 10-fold cross-validation on the whole dataset. The classification accuracy remains stable under the additional sampling methods.

**Table 1:** Performance metrics of Linear SVM classi er assessed by standard 10-fold cross validation, leave-one-outbreak-out validation and random undersampling methods

Sampling Methods	Accuracy	Precision-Chronic	Precision-Recent	Recall-Chronic	Recall-Recent	AUC
Standard 10-fold cross-validation	97.545%	98.105%	96.515%	98.446%	95.364%	96.905%
Leave-one-outbreak-out	96.075%	97.004%	91.0%	98.446%	83.5%	90.973%
Random undersampling	95.164%	96.328%	94.661%	94.155%	96.173%	95.164%

### Detection of transmission clusters

**Fig. 6:**
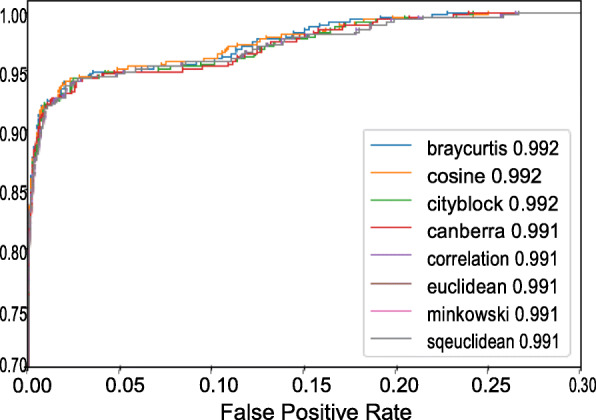
Performance of AUROC in detection of epidemiologically related pairs of populations with different distance metrices

The results of *k*-means, mini-batch *k*-means and hierarchical clustering models are shown in Table 2. In our experiments, agglomerative hierarchical clustering with ward linkage and euclidean distance between images demonstrated the best performance. Furthermore, we evaluated the accuracy of detection of epidemiologically related pairs. Two intra-host viral populations are considered to be related, if the distance between corresponding images is below a specified. threshold. ROC curves for the accuracy of detection of epidemiologically related pairs for different distance measures and thresholds are shown in Fig. 6. All distance measures produced consistent results, with AUC exceeding 0.99 for all of them.

**Table 2:** Performance metrics of various clustering methods

Clustering Method	NMI	homogeneity	completeness
*k*-means	0.986	0.994	0.978
Mini-batch *k*-means	0.985	0.992	0.978
Hierarchical	0.987	0.994	0.979

### Effect of image resolution

**Fig. 7:**
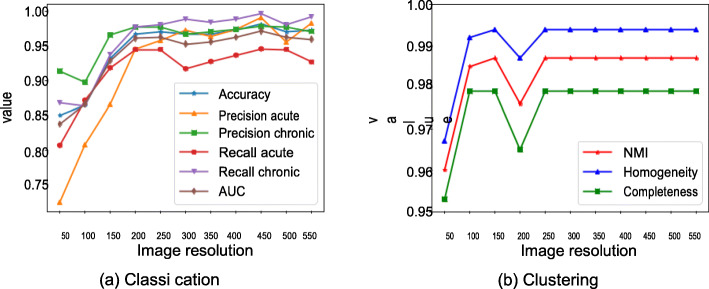
Performance metrics (Y-axis) of classification and clustering methods based on different image resolutions(X-axis)

All experimental results discussed above have been obtained using the default image resolution 480 × 480. We analyzed impact of image resolution on the classification and clustering performance. Resolution values varied from 50 × 50 to 550 × 550 with step size of 50. Fig. 7a shows the performance metrics of stratified. 10-fold cross-validation using LinearSVM model for detecting stage of HCV infections based on different image resolutions. Highest accuracy is achieved at the resolution 450 × 450, although the accuracy mostly saturates approximately after the resolution 300 × 300. Similar performance has been observed for agglomerative hierarchical clustering (Fig. 7b).

### Comparison with previous methods

A previously published model [32] classifies stages of HCV infection using one of the following 3 parameters: variant frequencies entropy, average position-wise nucleotide entropy and the average distance from viral variants to the most frequent variant of the population. In our data, AUC for these parameters was equal to ~81, ~66 and ~78%, respectively, while the proposed classifier based on image normalization yielded ~96.9% AUC.

We also compared clustering sensitivity and AUROC (of the inference of genetic relatedness between a pair of HCV samples) for our method and consensus-based approach (see e.g. [9, 33]) for the two population based methods VOICE and ReD proposed in [18]. The consensus-based method compares intra-host viral population using one representative sequence per population, which is most often the consensus sequence, while VOICE and ReD methods analyze whole quasispecies populations. Consensus algorithm achieves clustering sensitivity of 93.94% and AUROC (genetic relatedness) of 98.7%. ReD method achieves clustering sensitivity of 96.3% and VOICE method achieves clustering sensitivity of 98.2% and AUROC (genetic re-latedness) of ~99%. Image clustering method achieves sensitivity of 98.181% and AUROC of 99.2% which are higher values than consensus and ReD methods and has similar performance to the VOICE algorithm.

## Discussion

The sequence-image normalization method described here provides a way to transform genomic data into image data which can be directly employed by machine learning methods. The proposed preprocessing method was specifically designed to addresses multiple challenges that currently impede applications of machine learning and deep learning methods to viral studies. These challenges could be thematically classified as follows:

### Challenges associated with technological limitations

High-throughput sequencing technologies are prone to errors and biases, which may significantly affect viral data. Indeed, frequencies of minor viral variants are often comparable with the level of sequencing noise; however, such variants should not be simply discarded based on some frequency threshold, since often they are the ones responsible for transmission, immune escape or therapy failure [3, 5, 6, 34–36]. Presence of sequencing errors introduces noise to data and produces outlier viral variants, which negatively affect the quality and accuracy of machine learning classifiers.

Another important problem is sampling and sequencing bias resulting in significant irregularities in the number and length of viral sequences from different infected individuals. If classifiers capture these artificial differences as significant associations, it may result in overfitting and decline of accuracy. Thus, application of machine learning to heterogeneous viral population data should be preceded by a preprocessing step to eliminate these irregularities via normalization procedure. However, selection of an appropriate normalization approach is challenging. For instance, if we use text classification techniques for preprocessing, difference in the number of sequences among different files needs to be controlled either by truncation or padding. This preprocessing, however, causes data loss (in case of truncation) or introduces irrelevant data (in case of padding). An optimal preprocessing method should not introduce such issues.

### Challenges associated with feature selection and feature extraction

Before applying machine learning methods to classification of heterogeneous viral populations, genomic data should be mapped into the euclidian space R^n^. It is usually achieved by identifying numerical features that are relevant to the problem under consideration. They can include various diversity measures [32], popula-tion genetic parameters [37], physico-chemical properties [16] and other parameters specifically tailored to particular problems. These features are generally identified in consultation with domain experts. Selection of the most relevant features is daunting and resource-consuming. A role of feature selection in determining classification performance is paramount. Selection of a limited number of features from certain domains inevitably results in loss of information, while increase of feature space dimensionality increases risk of overfitting and compromises the algorithm’s scalability.

An optimal feature selection method should be able to capture the entire population structure using a relatively simple and easily contractible data representation. Furthermore, it should use a standard universal data format, which has a fixed number of features and is applicable to different problems. Since genomic data is essentially a textual information, it is tempting to utilize well-developed machinery from the text classification domain [38, 39] for the purpose of construction of such representation. Viral populations could be mapped to a euclidian spase using word2vec approaches [40], and classified using various available deep learning models [38, 39]. However, application of text processing approaches to viral research could be impeded by several factors. Since they are based on deep neural network models with large numbers of hyperparameters, it requires large annotated datasets to train these models. However, in molecular epidemiology, the amount of available training data is usually limited in comparison with the text processing domain. The datasets of several hundred intra-host viral populations analyzed in this paper are typical in this context. Although, word2vec or document embedding methods can be directly employed, it is challenging to train a model to get a higher classi classification performance. Furthermore, since viral haplotypes are unique, the trained model could overfit the data.

### Challenges associated with data comparison

Clustering of intra-host viral populations requires an inter-population distance measure, which takes into account complex population structures. It has been shown that among simple alignment-based population distance measures, the minimal distance between population variants allows to achieve the highest clustering accuracy [41]. However, this measure is sensitive to noise and presence of outliers, and does not take into account the whole population structure. Recently, several simulation-based and network-based distance measures have been proposed [18, 19], which overcome above-mentioned limitations at the cost of lesser scalability. Thus, the universal, accurate and efficiently computable inter-population distance measure, which takes into account complex population structures still has to be developed.

Our proposed preprocessing method converts the viral population genomic data sampled by NGS into a scaled image. Irregularities in the data are thus handled by generating a fixed size image. The number of features in this case remains same. Therefore, it can be directly used for machine learning applications without any explicit feature selection methods. High accuracy of machine learning classification and clustering techniques based on image representation applied to several molecular epidemiology tasks signifies validity of our approach. The case of infection staging is particularly illustrative. Previous studies demonstrated that diversity of intra-host viral populations often increases with progression of HCV infection [28, 32, 37]. In addition to immune escape, which is usually responsible for the diversity increase, complex adaptation mechanisms get engaged during intra-host HCV evolution, such as antigenic cooperation [6], which may result in increase of negative selection and selection of viral variants with particular properties, allowing HCV to survive in host environment for prolonged periods of time [17, 42–45]. The major features of such evolutionary processes include (but not limited to) low DN/DS ratio, skewed distributions of physico-chemical properties and presence of particular sequence motifs [16, 17, 37]. These and other features can be taken into account by inclusion of the features based on various genomic and biochemical parameters into machine learning classifiers. However, most of them are already implicitly included into the image representations, and thus are taken into account when the image-based classifiers are trained. It allowed us to achieve a high classification accuracy. In future work, sequence image normalization machinery can be applied to other challenging problems in viral genomics, such as detection of co-infections and prediction of drug resistance and therapy outcome.

## Conclusions

Here, we propose a novel method for generation of a fixed set of features representing heterogeneous viral populations, which is widely applicable for various classification and clustering tasks addressed by machine learning. The method converts sequence data into fixed-size images, thus reducing several issues associated with comparison of viral populations by machine learning methods. Simplicity of the sequence image normalization method allows for a robust conversion of genomic data into numerical data. Image data also help in visualization of genomic data. Experimental results demonstrate that the proposed method can be successfully applied to different problems in molecular epidemiology and surveillance of viral diseases. Simple binary classifiers and clustering techniques applied to the image data are equally or more accurate than other models.

## Data Availability

The data used in this paper has been published in [13] and partially in [8]. It can be shared by reasonable request. The developed software is freely available at https://github.com/compbel/SequenceImageNormalization
